# The impact of logistic environment and spatial spillover on agricultural economic growth: An empirical study based on east, central and west China

**DOI:** 10.1371/journal.pone.0287307

**Published:** 2023-07-26

**Authors:** Xuelan Li, Jiyu Jiang, Javier Cifuentes-Faura

**Affiliations:** 1 School of Economics and Management, Anhui Agricultural University, Hefei, Anhui, China; 2 School of Management, Anhui Science and Technology University, Bengbu, Anhui, China; 3 Faculty of Economics and Business, University of Murcia, Murcia, Spain; Hebei Agricultural University, CHINA

## Abstract

**Background:**

To vigorously promote the integrated development and mutual adaptation of agriculture and logistics is an important way to realize agricultural modernization and rural revitalization. Along with the policy support of agricultural industry chain and the steady rise of the demand for agricultural products market, the total amount of agricultural product logistics continues to increase, and the growth rate remains stable. With the booming development of "Internet + agriculture" and e-commerce platform, agricultural logistic market welcomes a new round of development opportunities, reaching several trillion yuan. Compared with the developed countries, our agricultural product logistics is still far behind. At present, only 15% of vegetables and fruits and 30% of fresh meat have professional logistic transportation, while most of the rest are still in the state of local and primitive. The gap of logistic environment construction and logistic elements determines the difference in final benefit of agricultural products. The purpose of this study is to find out the influence of logistic elements on regional agricultural economic growth, and whether the influence between neighboring regions presents "the same prosperity", "the same loss" or "sharing weal and woe".

**Methods:**

Based on the panel data of the statistical yearbook of 31 provinces in China from 2005 to 2020, the spatial Durbin model was constructed under the spatial weight matrix of economic distance and economic geographical distance to conduct empirical analysis, and the internal factors of logistics industry, factor spillover effect and its impact on agricultural economic growth were studied.

**Results:**

Results showed that: (1) considering economic distance factor, the spatial coefficient of the time-fixed effects model passed the significance test in eastern China. Considering economic geographic distance factors, the individual and double fixed effect models passed the significance test in central China, and all models passed the significance test in western China. (2) From the perspective of logistic infrastructure, AVLFA, HM, TN and RM had a positive effect on the growth of agricultural economy in eastern China, but LIAV is on the contrary. AVLFA had a positive effect on agricultural economic growth, but TN was on the contrary in central China. In western China, LIAV and TN promoted agricultural economy while HM and RM held back it. From the perspective of the volume of logistics activity, both eastern and central regions did not pass the significance test, but FA was tested by the double fixed effect model and showed negative in western China. From the perspective of control variables, FU, AO and PT all promoted agricultural economy in eastern China, and FU and AO did the same in central and western China. PT was invalid in central regions and hindered agricultural economy in western China, which was different.

**Conclusion:**

From the perspective of spatial spillover effect decomposition, the eastern region presents "one prosperity and all prosperity, and sharing weal and woe", while the central and western regions present "one prosperity and all prosperity, and one lost and all lost". At last suggestions as formulating the overall plan for the development of regional logistics, paying attention to regional differences and promoting coordinated development of logistics and agriculture in light of local conditions, and paying attention to the spatial spillover effect of elements were put forward.

## Background

**Fig 1:**
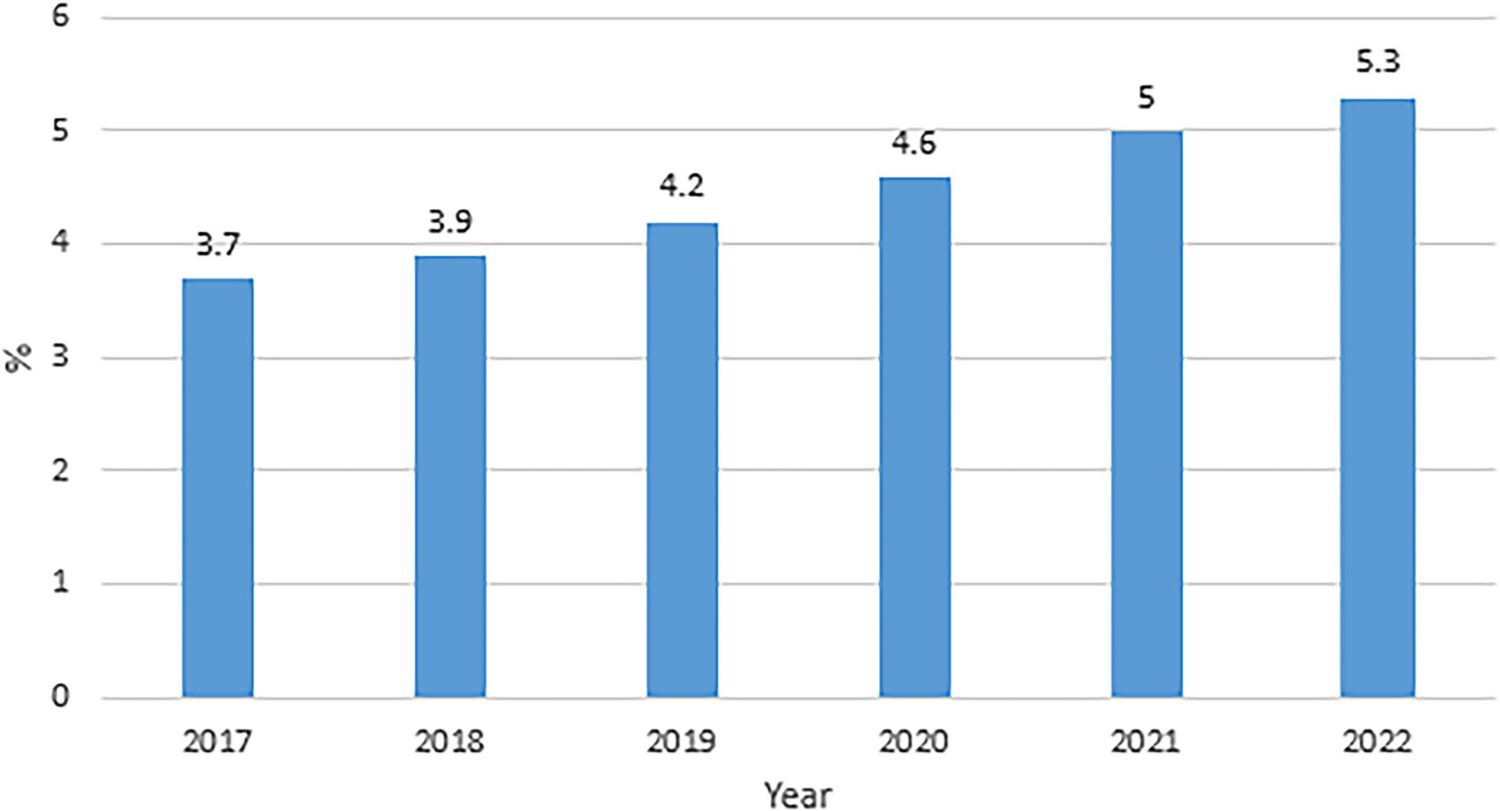
Growth of agricultural logistics from 2017 to 2022 in China.

China is a major producer and consumer of agricultural products. It has 18.7% of the world’s population, but half of the world’s pork consumption, 49.7% of the world’s vegetable consumption, and 34.4% of the world’s fish and seafood consumption. In the past few decades, China’s agricultural development and reform have achieved successful experience, mainly relying on rural institutional innovation, agricultural technology progress, agricultural market reform and agricultural input. Agricultural logistics, as an important support to achieve the goal of agricultural modernization, is related to the fundamental interests of farmers. The purpose is to use modern logistic means to promote the added value of agricultural activities and agricultural products, reduce production and circulation costs, improve market reaction speed, agricultural product delivery quality and customer satisfaction level, and then improve the overall efficiency of agricultural supply side to effectively solve "agriculture, rural areas and farmers" problems. Along with the policy support of agricultural industry chain and the steady rise of the demand for agricultural products market, the total amount of agricultural product logistics continues to increase, and the growth rate remains stable (as shown in Fig 1). With the booming development of "Internet + agriculture" and e-commerce platform, agricultural logistic market has welcomed a new round of development opportunities, reaching several trillion yuan [1]. The No.1 document of Chinese Central Committee in 2021 especially proposed that the construction of agricultural modernization and agricultural product logistics development will be accelerated in the "14th Five-Year Plan" period. In 2023, No.1 document of Chinese Central Committee clearly pointed out to speed up the improvement of e-commerce and logistics distribution system in counties and villages.

In developed countries, the loss rate of fruits and vegetables is generally controlled below 5%. In the United States, the loss rate of fruits and vegetables from field to table is only 1%-2% because the whole industrial chain of agricultural products is supported by cold chain logistics. Compared with the developed countries, our agricultural product logistics is still far behind. At present, only 15% of vegetables and fruits and 30% of fresh meat have professional logistics transportation, while most of the rest are still in the state of local and primitive [2]. The gap of logistic environment construction and logistic elements determines the difference of final benefit of agricultural products. Similar to the difference of economic growth caused by the difference of regional resource endowment, the driving effect of logistic factors on agricultural economic growth is also different due to the development levels of regional logistics industry. At the same time, the flow and spillover effects of inter-regional logistics factors make agricultural economic growth inextricably linked with the logistics industry in neighboring regions. Rational distribution of agriculture and logistics makes the two industries adapt to each other and develop together, and gives play to the driving role in areas with better integration, which is of great significance to the high-quality development of agriculture. From the current situation of agricultural economic development in China, there is an obvious gap between the eastern, central and western regions. The development of the western region lags behind that of the central and eastern regions, and the inter-provincial development gap is larger than that of the central and eastern regions [3]. Then, whether the logistics industry elements and their changes caused the agricultural economy of regions and adjacent areas "one prosperity and all prosperity" and "one lost and all lost"? What are the specific effects? Are the characteristics consistent across regions?

## Literature review

Scholars have explored the important influencing factors of regional agricultural economic growth. Inumula et al. conducted an empirical test on energy consumption and agricultural economic growth through co-integration test and vector error correction model, and the results showed that there was a long-term correlation between energy consumption and agricultural economic growth [4]. The substitution of biofertilizers for fertilizers has boosted agricultural economics and had a positive impact on environmental protection [5–8]. In the study on agricultural exports and agricultural economic growth, Seok and Moonconducted a group study on developed countries [9], Ahmed and salam, Bulagi et al., and Ramphul conducted a case study on developing countries [10–12]. Koondhar et al. (2021a) found that carbon emission has a significant negative impact on agricultural economy using data from 1985 to 2018, by analyzing ARDL and VECM along with novel DYARDL simulations [13].

The academic circle has reached a general consensus on the role of logistics industry in agricultural economic growth. A study analyzed the connection between agricultural logistics and agricultural economic growth and proposed several ways to accelerate agricultural economic growth by improving the level of agricultural logistics, such as regional agricultural logistics development planning consistent with the new rural planning, the construction of multiple new agricultural logistics systems, the development of oriented characteristic agricultural logistics, a wider range of circulation of agricultural products [14]. Some scholars suggested that the increase of logistics costs, especially transportation and storage costs, would cut into the growth of agricultural productivity. Agricultural economic development can be promoted by considering sustainability concepts, adopting simple and multimodal routes, normally evaluating logistics performance [15]. Another study proposed from the perspective of industrial symbiosis that only the coordinated and interactive development of agriculture and logistics could achieve co-prosperity, which was the core issue facing by the current development of the two sides [16]. Green logistics of agricultural products is an important path to develop low-carbon economy and can effectively promote sustainable agricultural development [17]. Green reverse logistics technology has an important impact on the operational efficiency and sustainable competitive advantage of agricultural entrepreneurial marketing enterprises [18]. Cold chain logistics is not only vital for maintaining the quality and safety of fresh products and reducing losses, but also provides important support for helping farmers increase their incomes, thereby contributing to rural revitalization [19].

Another focus of scholars’ attention is the development of agricultural logistics. The efficiency of agricultural logistics in China was estimated and it was found basically stagnant from 2003 to 2011 [20], with the highest in the eastern region, followed by the central region and the lowest in the western region. Insufficient capital investment in logistics is the main obstacle to the development of agricultural product logistics in China. The logistics efficiency of agricultural products in the central region of China showed that the fixed asset investment in the logistics industry was the main influencing factor, while energy consumption, the number of employees and carbon emissions of agricultural product logistics industry were secondary influencing factors [21]. Some pointed out that Agricultural product logistics is facing problems such as weak infrastructure construction, lack of agricultural insurance and high operating costs, and put forward countermeasures such as policy guidance, insurance entry, new technology utilization and business model innovation [22]. The society did not pay enough attention to agricultural logistics, the fruit and vegetable wholesale market, farmers’ market and other similar places that agricultural logistics mainly relies on had not formed logistics service system [23]. Some scholars proposed that the threshold and quality of foreign investment introduction should be continuously raised for the eastern regions of China, which were with large stock, while for the underdeveloped provinces in the central and western regions, due to their insufficient opening to the outside world, they needed to improve their location conditions and focused on improving infrastructure to attract more foreign investment [24]. Some scholars proposed the direction of innovation and upgrading of agricultural product logistics from the perspective of logistics information management and organization [25]. The application of agricultural logistics information system in the context of supply chain management could be a strategy to ensure the success of optimal distribution of agricultural products [26].

**Fig 2:**
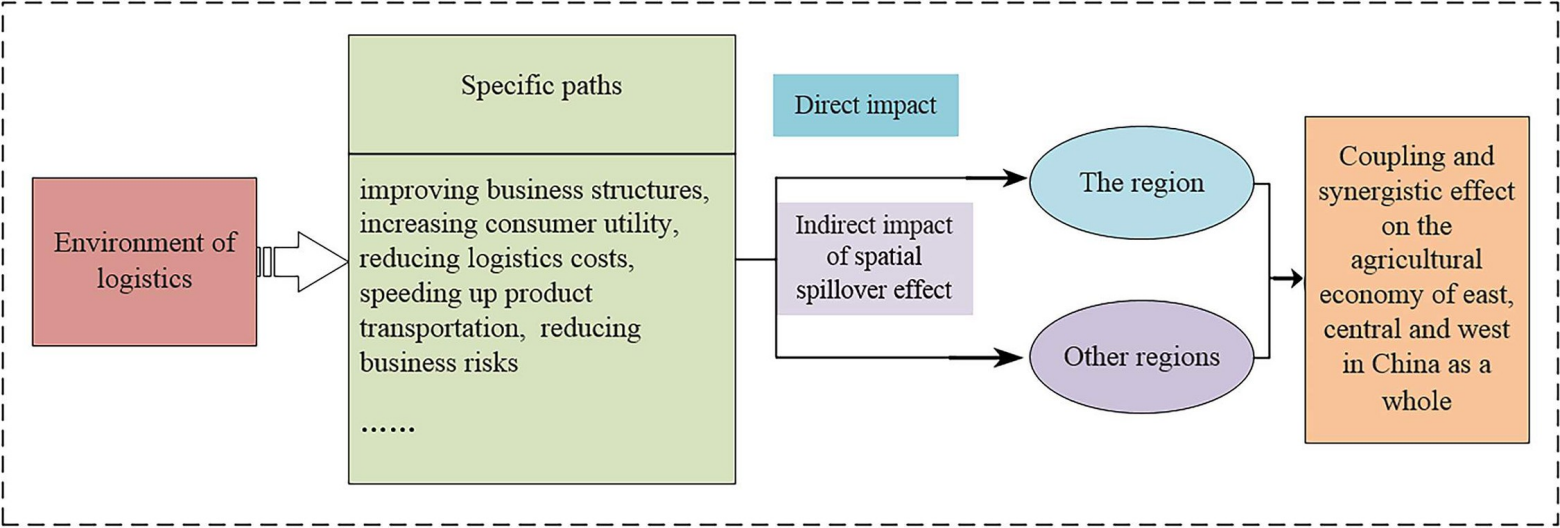
The logical framework diagram.

Through sorting out relevant research results, it is found that the growth of agricultural economy is closely related to various influencing factors, but there are few studies that combine agricultural factors with logistics factors, especially considering the influence of inter-regional spatial spillover effect on agricultural economic growth. We believe that the environment of logistics will have a direct impact on the agricultural economic growth of the region through specific paths such as improving business structures, increasing consumer utility, reducing logistics costs, speeding up product transportation, and reducing business risks, and will further have an indirect impact on the agricultural economy growth of other regions through spatial spillover effects, which in turn will have a coupling and synergistic effect on the agricultural economies of east, central and west in China as a whole. The logical framework diagram is shown in Fig 2. Therefore, this paper builds a spatial econometric model based on the panel data of 31 provinces in China, conducts an empirical study on the driving effect of logistics industry elements and their spatial spillover effects on agricultural economic growth, and comprehensively grasps the influence of logistics industry elements from within the region and other regions on agricultural economic growth, and on this basis, relevant countermeasures and suggestions were put forward.

## Methods

Economic activities always unfold in dimensions of both time and space, so it is necessary to consider the degree of correlation of spatial units of economic phenomena. In view of the fact that traditional econometric methods such as Granger causality test, co-integration theory and coupled cooperative degree model do not consider the spatial differences between research objects, spatial metrology methods are used in this research, modifying the regression analysis through an exogenous spatial weight matrix to make the regression results more reasonable. The analysis process mainly includes the following steps:

### Construct spatial weight matrix

The spatial weight matrix is used to describe the relationship between adjacent objects in space. As an important tool to study the influence relationship between different objects from the perspective of space, the physical distance between objects is usually used to reflect the spatial relationship, that is, to determine the influence degree according to the distance. China has a vast territory, and there are significant differences in regional agricultural economic development levels, referring to the research methods of relevant literature, this paper uses economic distance space matrix and economic geographic distance space weight matrix in the empirical process.

### Judge the spatial autocorrelation of variables

According to the first law of geography, things close to each other are more closely related than things far apart. Exploratory spatial analysis is the basis for determining the degree of spatial agglomeration and subsequent spatial econometric analysis. Global Moran’s I index and Local Moran’s I index are used to test the global and local spatial autocorrelation of variables. Global Moran’s I Formula is as follows:

$$I=\frac{\sum_{i=1}^{n}\sum_{j=1}^{n}W_{ij}\left(X_{i}-\bar{X}\right)\left(X_{j}-\bar{X}\right)}{S^{2}\sum_{i=1}^{n}\sum_{j}^{n}W_{ij}}$$ (1)

Among them: *X*_*i*_ and *X*_*j*_ are the index values of each region, $\bar{X}$ is the mean value of the index, *S*^2^ is the variance, *W*_*ij*_ is the spatial weight matrix, and *I* is the total number of the research area. Global Moran’s I<1, the size of which indicates the strength of the correlation. *I* > 0 means the space is positively correlated, *I* < 0 means the space is negatively correlated, and *I* = 0 means the space is not correlated. Local Moran’s I essentially disperses the values of the global Moran index to each region, and its formula is as follows:

$$I_{i}=\frac{\left(X_{i}-\bar{X}\right)}{S_{i}}\sum_{i=1}^{n}W_{ij}\left(X_{i}-\bar{X}\right)$$ (2)

If *I*_*i*_ > 0, the region is divided into HH (high) and LL (low and low) types. The region *I*_*i*_ < 0 was divided into HL (high and low) and LH (low and high) types. HH (high) indicates that highly correlated regions are surrounded by highly correlated regions, LL (low and low) type means that the low correlation area is surrounded by the low correlation area, HL (high and low) type means that the high correlation area is surrounded by the low correlation area, LH (low and high) means that the low correlation area is surrounded by the high correlation area.

### Build a spatial econometric model

Spatial econometric models can be built after the global and local Moreland indices of space exploration analysis pass the test, which commonly include spatial lag model, spatial error model and spatial Dubin model. Spatial lag model considers the spatial spillover of explained variables, spatial error model considers the spatial spillover of explanatory variables, and Spatial Durbin Model (SDM) considers the influence of explanatory variables, control variables and spatial spillover effect simultaneously. That is, the model can not only consider the influence of agricultural factors and logistics factors on agricultural economic growth, but also consider the influence of spatial spillovers. The spatial spillover effect can be divided into direct effect, indirect effect and total effect. The direct effect is the influence of the logistics industry on the growth of regional agricultural economy in a certain region, while the indirect effect is the influence of the logistics industry in this region on the growth of agricultural economy in other regions, namely, spillover effect. The total effect is the superposition of the two. On this basis, the spatial Durbin model is constructed, and its basic form is as follows:

$$Y=\rho WY+X\beta +\theta WX+\alpha l_{n}+\varepsilon$$ (3)

Among them, Y represents the explained variable, X stands for explanatory variable and control variable, *ρ* stands for spatial autocorrelation, W is the constructed spatial weight matrix; WX and WY respectively represent the logistic industry related factors of the explanatory variable and the spatial lag term of the agricultural economic growth of the explained variable; β and θ represent the regression coefficient, and ε is the error term.

It is important to note that the difference-in-differences model and the fixed effects model are two methods commonly used for spatial Durbin models. Among them, the difference-in-differences model is a method used for the baseline characteristics of the panel data. The model applies two differences to the panel data to eliminate the confounding time-invariant individual characteristics and the heterogeneity between the treated and untreated groups. Specifically, the model has the following three advantages: (1) it has higher controllability and reliability compared with the general regression model; (2) it can eliminate time-invariant individual characteristics and heterogeneity between treated and untreated groups, and obtain more accurate estimates of causal effects; (3) it has a clearer interpretation of the significance of asymptotic variables such as individual fixed effects. However, the model also has two drawbacks: (1) it has limited applicability to scenarios where the treated and untreated groups each contain a time series; (2) in some cases, it may mask changes in the characteristics of the treated and untreated groups. However, the fixed-effects model can address the drawbacks of the difference-in-differences model, and most importantly, it is easier to improve the model accuracy and controllability in the case of long time series. Combined with the data used in this paper (panel data, 31 Chinese provinces from 2005 to 2020, and belongs to long time series), the authors concluded that the fixed-effects model is more suitable for this paper.

### Statistical methods

#### Variables and data sources

According to the selection of variables in existing research literature [27, 28], and considering the reduction of rural population caused by urbanization, the main variable in this paper uses per capita index. Per capita agricultural added value (AAV) was taken as the explained variable of the measurement of regional agricultural economic growth. In order to eliminate the inflation factor, agricultural added value took 2004 as the base period, and the corresponding provinces were subtracted by the deflator, and the results were obtained after dividing by the number of township population in each year. Regarding the core explanatory variables. We refer to previous studies [29–31] to measure the indicators of the logistics industry in two dimensions: logistics infrastructure and logistics activity. Logistics infrastructure includes added value of logistics industry (LIAV), added value of logistics fixed assets (AVLFA), highway mileage (HM), railway mileage (RM) and TN (TN). Logistics activities include freight amount (FA) and freight turnover (FT). In order to ensure the comprehensiveness of the study and reduce the impact of omitted variables on the empirical study, power of agricultural machinery (PAM), fertilizer usage (FU), agricultural output (AO) and population of township (PT) were selected as the control variables in this paper, which is based on the practice of existing relevant studies [32–34]. Except the number of township population itself, all other variables are divided by the number of township population to obtain the average. All data are from China Statistical Yearbook. Due to the missing of some agricultural index data, the data panel is unified from 2005 to 2020.

#### Empirical analysis

The empirical process was carried out by STATA16 software. Firstly, the Moran’s I index of global space was calculated (the results are shown in Table 1). It can be seen that the Moran’s s I index of 2018, 2019 and 2020 were significant at the significance level of 0.05. The Moran’s I index in other years was significant at 0.01 level. Therefore, the spatial spillover effect of agricultural economic growth in China is obvious, which can be analyzed by spatial econometric analysis.

**Table 1:** Global spatial Moran’s I index of agricultural added value from 2005 to 2020.

year	2005	2006	2007	2008	2009	2010	2011	2012
Moran’s I	0.147	0.156	0.181	0.174	0.187	0.183	0.192	0.176
z_value	4.560	4.747	5.276	5.097	5.402	5.301	5.453	5.083
P_value	0.000	0.000	0.000	0.000	0.000	0.000	0.000	0.000
**year**	**2013**	**2014**	**2015**	**2016**	**2017**	**2018**	**2019**	**2020**
Moran’s I	0.159	0.143	0.127	0.111	0.109	0.085	0.068	0.053
z_value	4.644	4.243	3.829	3.460	3.428	2.858	2.463	2.096
P_value	0.000	0.000	0.000	0.001	0.001	0.004	0.014	0.036

**Fig 3:**
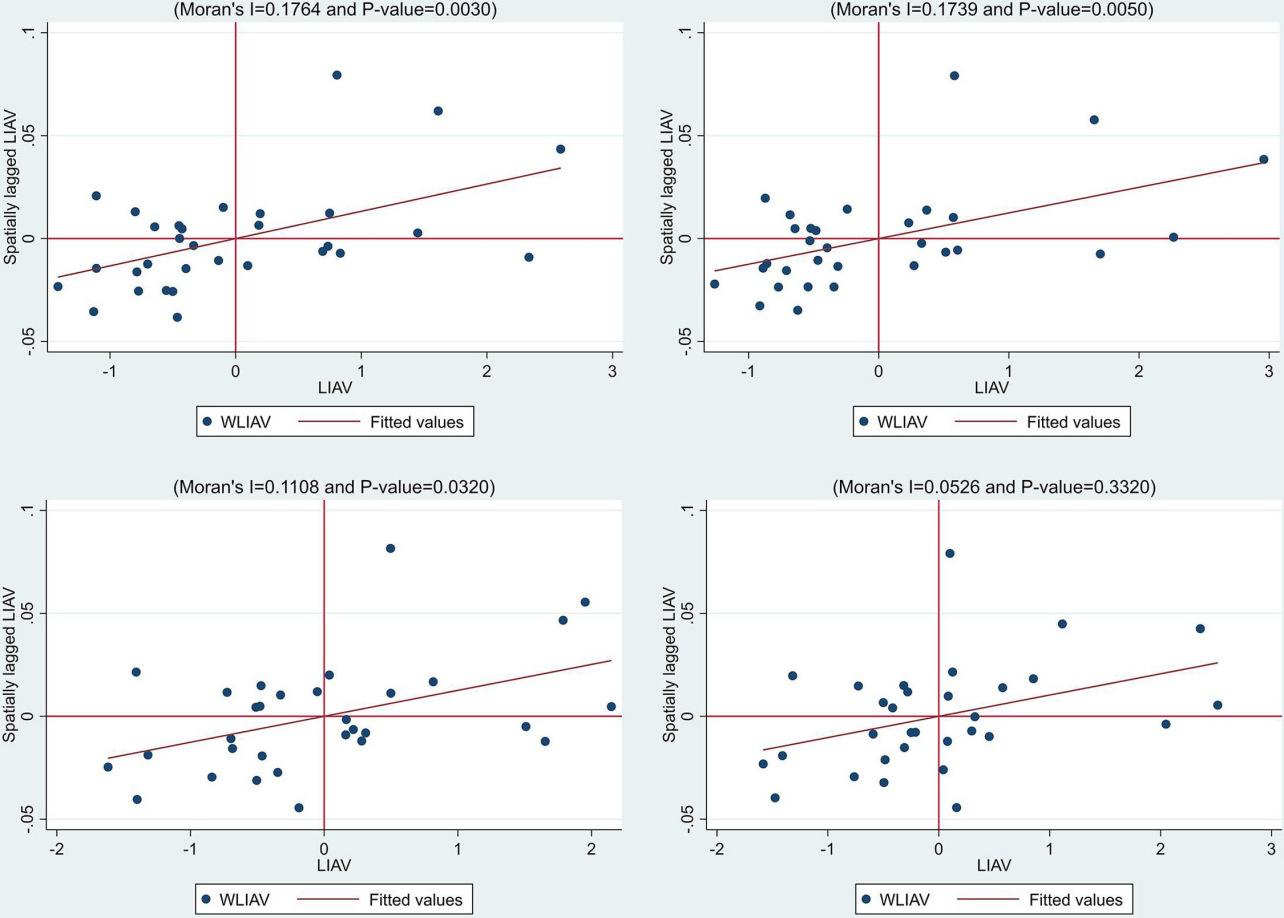
Local Moreland index scatter plots in 2008, 2012, 2016 and 2020.

The local Moran index of 2008, 2012, 2016 and 2020 was used to draw scatter charts (limited by space), as shown in Fig 3. It can be seen that most provinces are clustered in the first and third quadrants, namely HH and LL regions, and the local spatial spillover effect is obvious, which can be demonstrated by using the spatial Durbin model.

## Results and discussion

According to China’s current commonly used division of geographical regions, the whole is divided into three regions, namely, the eastern region: including 11 provinces and municipalities as Hebei, Liaoning, Shanghai, Beijing, Jiangsu, Zhejiang.Fujian, Shandong, Guangdong, Tianjin, and Hainan; Central region: including 8 provinces as Shanxi, Jilin, Heilongjiang, Hubei, Hunan, Henan, Anhui and Jiangxi; Western region: including 12 provinces and municipalities as Neimenggu, Chongqing, Sichuan, Guizhou, Yunnan, Tibet, Shaanxi, Gansu, Qinghai, Ningxia, Xinjiang and Guangxi, and three regions respectively constructed the spatial Durbin model. The natural environment and agricultural resource endowment of different regions vary greatly, and there may be omitted variables that do not change with time, so the adoption of fixed effect model is more common. At the same time, Hausman test found that Prob> CHI2 = 0.0000, which rejected the random effect model at the significance level of 0.05, so the fixed effect model was adopted. On this basis, economic distance matrix and economic geographic distance matrix are respectively used to conduct empirical demonstration of spatial Durbin model. Tables 2–4 are the empirical results of eastern China, central China and western China respectively. In each table, (1)—(3) are the empirical results of individual fixed effect, time fixed effect and double fixed effect under the spatial weight matrix of economic distance, and (4)—(6) are the empirical results under the spatial weight matrix of economic geographical distance. In the empirical analysis, if the double fixed effect model is adopted, the empirical results of the double fixed effect model will be the main one, and the single fixed effect model will also be considered. Tables 5–7 show the empirical results of spillover effect and spillover effect decomposition of logistic factors in eastern, central and western China respectively.

**Table 2:** Regression results of spatial Durbin model in eastern China.

	Model (1)	Model (2)	Model (3)	Model (4)	Model (5)	Model (6)
Main						
LIAV	-0.976***	-0.652**	-1.116***	-0.239	-0.403*	-0.301
AVLFA	0.566***	0.460***	0.438***	0.217**	0.348***	0.332***
HM	-80.70	99.80**	175.0***	134.9***	146.2***	136.6***
RM	3.773***	4.151***	4.140***	2.254***	1.455**	1.382**
TN	8558.6***	6384.4***	2180.0*	7829.9***	7153.3***	7518.4***
PAM	-0.0545**	0.0158	-0.0237	-0.0166	-0.0192	-0.0179
FU	2.173*	8.732***	10.11***	7.046***	9.539***	8.831***
AO	0.130*	0.163*	0.123*	0.0212	0.00587	0.00644
PT	0.0000669*	0.000132*	0.000211***	-0.000145**	-0.000124	-0.000156*
S-rho	0.0826	0.236**	0.0330	0.706	-1.660	-1.563
V-sigma2_e	0.00798***	0.00413***	0.00281***	0.00314***	0.00242***	0.00260***
N	176	176	176	176	176	176

t statistics In parentheses:

* p<0.05

**p<0.01

***p<0.001

**Table 3:** Regression results of spatial Durbin model in central China.

	Model (1)	Model (2)	Model (3)	Model (4)	Model (5)	Model (6)
Main						
LIAV	-1.373	0.135	-0.957	1.891	2.192*	1.725
AVLFA	0.0666***	-0.114	-0.103	0.0402*	0.0316	0.0667*
HM	-30.19	-25.00	-44.41	-66.37	-151.3**	7.352
RM	18.01*	7.852	4.161	12.66	8.919	19.32
TN	-2582.1*	-3970.3*	-3815.8*	-6372.4***	-7925.7***	-5848.3**
FU	0.110	1.752***	1.610**	-0.0888	0.389	-0.838
AO	0.251***	0.335***	0.225**	0.507***	0.482***	0.505***
PT	-0.000015	0.000145	0.0000860	0.0000227	0.000532***	0.0000670
S-rho	-0.0319	0.167***	0.0569	7.760*	8.382	7.820*
V-sigma2_e	0.00472***	0.00545***	0.00390***	0.00917***	0.00822***	0.0101***
N	128	128	128	128	128	128

t statistics in parentheses:

* p<0.05

** p<0.01

*** p<0.001

**Table 4:** Regression results of spatial Durbin model in western China.

	Model (1)	Model (2)	Model (3)	Model (4)	Model (5)	Model (6)
Main						
LIAV	0.467***	0.414***	0.416***	0.423***	0.425***	0.545***
HM	-0.264***	-0.171*	-0.194***	-0.210***	-0.189**	-0.229***
RM	-0.0437**	-0.0594***	-0.0487**	-0.0405*	-0.0542***	-0.0246
TN	0.207*	0.211***	0.133*	0.0785	0.181***	0.151*
FA	-0.0859	-0.0484	-0.0919	-0.0874	-0.0547	-0.314***
PAM	-0.018	-0.154	-0.118	-0.122	-0.207*	-0.451***
FU	0.538***	0.567***	0.576***	0.561***	0.571***	0.519***
AO	0.181*	0.138***	0.166***	0.127**	0.117**	0.118**
PT	0.0561	-1.205***	0.0939	0.0880	-1.191***	-1.311***
S-rho	-28.98**	-44.14*	-45.37**	-13.38*	-12.17*	-20.28*
V-sigma2_e	0.00797***	0.00555***	0.00738***	0.00746***	0.00543***	0.00413***
N	192	192	192	192	192	192

t statistics in parentheses

*p<0.05

**p<0.01

***p<0.001

**Table 5:** Spatial spillover effect decomposition in eastern China.

Index	LR_Direct	LR_Indirect	LR_Total	Ratio of LR_Indirect	Model Type
LIAV	-0.588**	0.777**	0.189	0.4308	(2)
AVLFA	0.449***	-0.148	0.302*	0.2479	(2)
HM	142.0***	-129.2*	12.77	0.4764	(4)
RM	1.919***	-12.69***	-10.77***	0.8686	(4)
TN	7394.9***	4113.2	11508.1***	0.3574	(6)
FU	8.831***	1.715	10.55***	0.1626	(2)
AO	0.187**	0.375**	0.562**	0.6673	(2)
PT	0.000121	-0.000116	0.00000500	0.5063	(2)

t statistics In parentheses:

* p<0.05

**p<0.01

***p<0.001

**Table 6:** Spatial spillover effect decomposition in central China.

Index	LR_Direct	LR_Indirect	LR_Total	Ratio of LR_Indirect	Model Type
RM	18.33	10.36	29.15**	0.3554	(6)
TN	-5472.5*	-2217.8	-7690.2*	0.2884	(4)
PAM	-0.0394	0.325**	0.285**	0.8919	(4)
FU	-0.572	1.598	-0.882***	0.7364	(4)
AO	0.660***	-0.882***	-0.222	0.5720	(4)

t statistics In parentheses:

* p<0.05

**p<0.01

***p<0.001

**Table 7:** Spatial spillover effect decomposition in western China.

Index	LR_Direct	LR_Indirect	LR_Total	Ratio of LR_Indirect	Model Type
LIAV	0.440***	1.290***	1.730***	0.7457	(6)
AVLFA	-0.0244	0.104	0.145*	0.7172	(3)
HM	-0.204**	-0.330**	-0.534***	0.6180	(6)
RM	-0.0258*	0.0196	-0.00618	0.4317	(6)
TN	0.136*	0.208	0.344	0.6047	(6)
FA	-0.00928	-0.635*	-0.644*	0.9860	(2)
FT	-0.0352	0.170	0.135	0.8285	(2)
FU	0.507***	0.311	0.818	0.3802	(6)
AO	0.109**	0.0772	0.186	0.4151	(6)
PT	0.0663	0.465**	0.532**	0.8741	(4)

t statistics In parentheses:

* p<0.05

**p<0.01

***p<0.001

### (1) Eastern region

From the regression results of economic distance spatial weight matrix and economic geographic distance spatial weight matrix, they are generally consistent, and the regression results are relatively robust. Under the economic distance weight matrix, the spatial coefficient of the time-fixed effects model (S-rho) passed the significance test. It indicated that there were time differences in the effects of influencing factors when considering the economic factors. Logistics infrastructure related indicators as LIAV, AVLFA, HM, RM and TN all had an impact on the growth of agricultural economy. The spatial influence coefficients of AVLFA, HM, RM and TN were positive, indicating that logistics infrastructure and agricultural economy in eastern China had a high degree of mutual adaptation, and better integrated development had been realized. In this area, there are many plains, the highway network is in all directions, and the truck has relatively few unsafe factors, the logistic cost is low and the benefit is good. The volume of import and export trade in the eastern coastal cities is large, mainly relying on railway transportation of bulk goods. Dense population and developed industry and commerce also give strong driving force for railway development. LIAV’s influence coefficient was negative, indicating that the increase of the added value of logistics industry did not lead to the increase of the added value of agriculture. This might be due to insufficient optimization of resource allocation and output in logistics industry. The volume of logistic activities can reflect the demand of regional logistics industry, which is usually measured by freight volume, cargo turnover, etc. FA and FT in the eastern region did not pass the significance test, indicating that the correlation between logistics activity and agricultural economic growth was not obvious. This might be due to the superior geographical location, strong technical force and solid industrial foundation of the region. Powerful industrial provinces as Guangdong, Jiangsu, Shandong, Zhejiang, Hebei and Fujian are all located in the eastern region [35], and agricultural products account for a relatively small proportion in the commodity transportation structure.

Control variables FU, AO and PT passed the significance test, and indicated that they had a significant promoting effect on the agricultural economic growth. FU in eastern China has decreased in recent years, which could also significantly promote agricultural economic growth. On the one hand, it was related to the "Zero growth action of chemical fertilizer use" proposed by the state in 2015, and on the other hand, it might be related to the intensity and effect of promoting environmentally friendly technologies in eastern China, which has passed the inflection point of environmental Kuznets curve, similar to the research conclusion of some scholars [36]. In 2019, rural online retail sales in the eastern region accounted for 78.6 percent of the country’s total. The central and western accounted for only 12% and 9.4%, respectively. In addition, the growth rate of online retail sales in rural areas in eastern China still ranked first, at about 19.8%. The new marketing model has stimulated the enthusiasm of farmers and the output of agricultural products, and promoted the development of agricultural economy.

The decomposition results of spatial effects in eastern China were shown in Table 5. Most indicators passed the time fixed effects model test under the economic distance weight matrix, which was model (2). The direct effect coefficient of LIAV was negative, while the indirect effect coefficient of it was positive, indicating that over time, LIAV in provinces of eastern china blocked the growth of agricultural economy in local provinces, but promoted the growth of other provinces. The Bohai logistics circle with Beijing, Tianjin, Shenyang, Dalian and Qingdao as the center, the Yangtze River Delta logistics circle with Shanghai, Nanjing, Hangzhou and Ningbo as the center, the logistics circle including Xiamen and Fuzhou around the Taiwan Strait, and the logistics circle around the Pearl River Delta centered on Guangzhou and Shenzhen have been formed in the eastern part of China. The improvement of logistics industry in certain provinces has contributed to the convenience of transportation and the creation of value in the logistics circle. Meanwhile, local agriculture faces the fierce competition with products from other provinces and cities inside and outside the circle. The direct effect and total effect coefficient of AVLFA were positive, which indicated that AVLFA in eastern China could promote the growth of the agricultural economy in local provinces and the whole region.

Among the control variables, FU had a significant positive impact on the agricultural economic growth of the local provinces, and AO not only had a significant positive impact on the agricultural economic growth of the local province, but also on other provinces.

### (2) Central region

The empirical results of SDM in central China showed that the time effect fixed model passed the significance test under the economic distance weight matrix, the individual fixed and the double fixed effect model passed the significance test under the economic geographic distance weight matrix. Among the indicators related to logistics infrastructure, TN passed the significance test in the two models, and its coefficient was negative, which fully indicated that TN had a blocking effect on agricultural economic growth when economic geographical distance factors were considered. The problem of weak agricultural quality in less developed areas is prominent. When the railway network is accessible, siphon and corridor effect will be formed, resulting in the loss of capital, talents, enterprises and other resources, and areas lacking economic competitiveness will be marginalized. Correlation between volume of logistics activity and agricultural economic growth was not obvious, which was the same to eastern China.

Among the control variables, AO passed the significance test under the individual fixed, time fixed and double fixed effect models, and showed a positive correlation, indicating that it played a positive role in promoting agricultural economic growth when considering the economic geographical distance factors. FU passed the significance test under the time fixed and double fixed effect models when considering the economic distance factor, and showed a positive correlation, indicating that this factor also played a positive role in promoting agricultural economic growth in central China.

The decomposition results of spatial spillover effects in central China were shown in Table 6. Most indicators passed the significance test of the individual fixed effect model under the spatial weight matrix of economic geographic distance, which was model (4). Among the indicators of logistics infrastructure, the direct effect and total effect of TN were all negative, which indicated that TN in the certain provinces was an obstacle to the growth of agricultural economy in local places and the whole area. By 2020, Shandong, Guangdong, Hebei, Henan, Zhejiang, Jiangsu, Anhui, Sichuan, Yunnan and Liaoning ranked the top 10 provinces in terms of truck ownership, with more than one million trucks. Six of them were in the eastern region, only two were in the central region, ranking the fourth and the seventh, and the left two were in the west. The number of trucks in the central region was far behind that in the eastern region, and its role in long-distance transportation and cross-regional transportation was limited. RM passed the significance test of the double-fixed effect model under the spatial weight matrix of economic geographic distance, and the total effect of space spillover was positive, indicating that RM contributed to the overall regional agricultural economic growth. The central region is short of sea transport, and the radiation effect of road transport is limited, so railway transport makes a great contribution. The first transverse of the "three vertical and three horizontal" trunk railway network is the Longhai Channel connecting the east and west, which is also an important part of the Eurasian Land Bridge. The second is the main passage along the Yangtze River, which passes through Hubei and Anhui provinces in the central region. The third section is the Shanghai-Kunming Passage, passing through Jiangxi and Hunan provinces. The spillover effect of other indicators of logistics infrastructure dimension and volume of logistics activity were not obvious.

Among the control variables, the direct effect of PAM was not significant, while the indirect effect and total effect were significant and the coefficient was positive, indicating that PAM in certain provinces promoted the growth of the agricultural economy in other provinces and the whole region. In terms of agricultural machinery, the central region has more advantages than the eastern region. The per capita cultivated land area, production area and agricultural machinery investment output are all larger. The direct and indirect effects of FU were not significant, but the total effect was significant and negative. The fertilizer accounted for 40%-60% of the total increase in crop yield, but the fertilizer utilization rate was only 30%-40%. The extensive use of chemical fertilizers has caused harm and hidden danger to soil, water, atmosphere and food chain, which was not conducive to food safety and high-quality development of agriculture. The total effect of AO was not significant, but the direct effect is positive and the indirect effect is negative, which indicated that AO in individual province promoted the growth of agricultural economy in the local place and impeded it in other places. The possible reason is that the central region has Henan, Anhui, Hubei and other provinces with large grain output. Of the country’s top 100 agricultural products counties, 55 are in central China, 38 are in eastern China and only seven in western China. Central Region has formed a series of national well-known agricultural products brands such as Xianfeng white tea, southern Jiangxi navel orange, Tongling white ginger, Xinyang Maojian, and Shanxi dry land tomatoes. The competition of agricultural products between provinces is strong.

### (3) Western region

The empirical results in western China showed that the spatial coefficients (S-rho) of all models passed the significance test. Among the indicators related to logistics infrastructure, the LIAV, HM, RM and TN all passed the significance test. Coefficients of LIAV and TN were positive, indicating that these two indicators drove the growth of agricultural economy. HM and RM coefficients were negative, indicating that these two indicators inhibited the growth of agricultural economy. The western region also suffers from the corridor effect after the completion of roads and railways. The industrial development lacks internal core competitiveness, and there are bottlenecks in attracting and stabilizing talents and investment. It may also because that the western region occupies too much capital for railway and highway construction, which makes the development of agriculture and other industries uneven and slows down the overall economic growth rate. Compared with the eastern and central regions, the western region made the most efforts in railway and road construction. Among the indicators of logistics activity, FA has passed the double-effect model test under the spatial weight matrix of economic geographic distance, and its coefficient was negative. The results showed that FA retarded the growth of agricultural economy. The top 10 provinces in terms of freight volume are Anhui, Guangdong, Shandong, Zhejiang, Jiangsu, Hebei, Henan, Hunan, Shanxi and Guangxi, with only Guangxi in the western region. Compared with the eastern and central regions, the transport infrastructure in the western region is still a key factor restricting the development. The number of external transport channels is small and the capacity is insufficient, which restricts the transformation of regional resource advantages into economic advantages.

Among the control variables, PAM has passed the time fixed effect and double effect model test under the spatial weight matrix of economic geographic distance, and the coefficient is negative. The western region is still faced with problems such as low overall level of agricultural machinery and equipment and uneven level of mechanization between regions, and the structural supply of agricultural machinery and equipment suitable for applications in mountain areas is obviously insufficient. PT passed the significance test of time-fixed and double-fixed effect models under the weight matrix of economic geographic distance, and the coefficient was negative, indicating that the reduction of rural population and labor force had a negative impact on agricultural economy. In the past years, the urbanization process in the western region accelerated, and the rural revitalization development index rose steadily, but it obviously lagged behind the development of new urbanization. Under the economic distant weight matrix and economic geographic distance spatial weight matrix, FU and AO both passed the individual fixed effect, time fixed effect and double fixed effect model tests, and the coefficient was positive, which significantly promoted agricultural economic growth. China abolished the value-added tax on chemical fertilizer in 2001 and implemented agricultural subsidies nationwide since 2004, which stimulated the input and use of chemical fertilizer. The use of chemical fertilizer in western China increased rapidly. The main agricultural products in this area include grain, cotton, melons and fruits, etc. The economic benefits of fertilizer input are more significant. There are many kinds and quantities of characteristic agricultural products in western China, including cotton, grape and sugar beet in Xinjiang, sugarcane in Guangxi, tobacco leaf in Yunnan and Guizhou, and milk in Neimenggu, which account for a high proportion in China [25]. Their popularity and influence are prominent, and they are the main sources for stabilizing and improving farmers’ income.

The decomposition results of spatial spillover effects in western China were shown in Table 7. Most indicators passed the significance test of the double-fixed effect model under the spatial weight matrix of economic geographic distance, which was mode (6). Among the indicators related to logistics infrastructure, the spatial direct effect, indirect benefit and total effect coefficient of LIAV, and the total effect of AVLFA were positive, indicating these two indicators could promote the agricultural economic growth of local provinces and other ones. LIAV in the western region was the largest during the study period compared to the eastern and central regions. The spatial direct effect, indirect benefit and total effect coefficient of HM were all negative, and it had an obstructive effect on agricultural economic growth in both local region and other regions. It might be related to the complex geographical environment, variable climate and backward logistics infrastructure construction. In western China, frequent extreme weather and complex terrain increase transportation costs and lack of security of goods, posing a great threat to the quality of fresh agricultural products, squeezing the profit margin of farmers in other regions and increasing the purchase risk of consumers. RM and TN had opposite direct effects on the province, with RM negative and TN positive. The possible reason was that the input of human resources and infrastructure in the railway logistics system did not match the output of the final freight volume and freight turnover, and the return to scale presents a diminishing state. There was obvious redundancy in logistics input, resulting in great waste.

Among the control variables, the spatial direct effect of FU and AO in western China was obvious and positive, indicating that the two have played a significant role in promoting the growth of agricultural economy in this region. The indirect and total spatial spillover effects of PT were positive, indicating that it effectively promoted the agricultural economic growth of other provinces. The change of population in townships released demographic dividend and promoted the upgrading of industrial structure through employment and consumption.

## Conclusions and policy suggestions

### Conclusions

Considering regional economic factors, economic geographic factors, the spatial Durbin model was used to study the influence of logistics factors and spatial spillover effect on agricultural economic growth. Six sets of spatial Durbin regression models were constructed under two spatial weight matrices, and the effects of individual fixed, time fixed and double fixed were analyzed respectively. The results showed that the spatial agglomeration effect of provincial agricultural economic growth in China was obvious. However, there are differences in influencing factors in different regions. Overall, the western region had the most impacting indicators, followed by the eastern region and the central region, specifically:

1. The results of spatial exploratory analysis showed that: during the study period, the global Moran index was positive. Local Moran index showed that most provinces were concentrated in HH and LL regions, indicating the spatial agglomeration of agricultural economic growth. At present, China is in the promotion stage of agricultural industry agglomeration, but some provinces have entered the restriction stage of industrial structure upgrading [37].
2. Considering economic distance factor, the spatial coefficient of the time-fixed effects model passed the significance test in eastern China. Considering economic geographic distance factors, the individual and double fixed effect models passed the significance test in central China, all models passed the significance test in western China, which fully proved that logistic environment and logistics elements had an impact on regional agricultural economic growth.
3. The results of spatial spillover decomposition showed that the spatial spillover effect of logistic environment and logistic elements was obvious in eastern, central and western regions. It demonstrated the phenomenon of "one prosperity and all prosperity" and "sharing weal and woe" caused by different influencing factors in eastern china, "one prosperity and all prosperity" and "one lost and all lost" in central China, and "one prosperity and all prosperity" and "one lost and all lost" in western China.

### Policy suggestions

According to the results of the empirical analysis, we put forward some policy suggestions:

1. Promote the unbalanced development strategy of agricultural industry agglomeration and optimize the allocation of regional agricultural resources. Avoid resource mismatch or inefficient supply due to excessive agglomeration or blindness, which will lead to crowding effect. Promote the agricultural industry agglomeration from "quantity increase" to "quality improvement", and gradually form the overall distribution of rational agricultural industry development pattern with distinct regional characteristics.
2. The influencing factors of agricultural economic growth in eastern, central and western regions are different. We should pay attention to regional differences and promote coordinated development of logistics and agriculture in light of local conditions. The early application of new technologies in the eastern region has promoted the development and efficiency of the logistics system, so the industry is about to enter the stage of structural optimization [38]. The fixed assets, railway mileage and freight cars of logistics industry in eastern China have a significant impact on agricultural economic growth, so we should continue to strengthen the investment in logistics infrastructure and logistics intelligence. Strengthen the promotion of environmentally friendly agricultural technologies in central and western regions, eliminate blind and excessive fertilization, and increase farmers’ knowledge of scientific fertilization. While improving railway and highway networks in the western region, advanced agricultural production techniques should be used to promote the output and quality of agricultural products. The western region is still in the process of industrial accumulation and expansion. The introduction of new technology and industrial modernization could have a positive impact on the total factor productivity growth of the logistics industry. Central area should not only perfect railway network but also strengthen agricultural product competitiveness. At the same time, accelerate the logistics reform, give full play to regional advantages and form an organic link between the eastern and western regions [39].
3. Attention should be paid to the spatial spillover effects of all factors. We should attach importance to trans-regional cooperation, constantly establish and improve a modern logistics system, form a reasonable and efficient trans-regional logistics network, and strengthen the inter-provincial flow of agricultural and logistics factors. Formulate the overall plan for regional logistics development, and promote the integrated development of logistics industry and other industries in the region. For the eastern region, high-quality logistics personnel should be trained, and integrated cooperation between enterprises, governments and universities should be realized. Logistics service projects closely supporting local agricultural products should be jointly developed, playing a radiating role in driving other regions. The central and western provinces should avoid blind development. While expanding the total amount of logistics infrastructure, they should actively communicate with provinces with obvious spatial spillover effect, build cooperation platforms, and increase the "stickiness" of capital and talents.

### Research limitations and prospects

Actually, there are two limitations in this paper. First of all, due to the limitation of data, this study cannot obtain a longer research period, so it is unable to conduct a more in-depth analysis of influence the logistic environment and logistics elements on the growth of China’s agricultural economy. Secondly, in order to reduce the endogeneity problem caused by cross-section data, we choose panel data for global regression, which may ignore the special changes in different stages during this period. Therefore, the data can be further improved in the future research and a long-span database can be established. To explore the impact of the evolution process of logistics, an emerging industry, on agricultural economy, the data of different stages can be compared in sections and as a whole. At the same time, more diversified index systems and analytical frameworks can be constructed.

## Supporting information

S1 Appendix Data for the indicators from 2005 to 2020. (XLSX)
